# Impulsivity: present during euthymia in bipolar disorder? - a systematic review

**DOI:** 10.1186/2194-7511-2-2

**Published:** 2014-03-11

**Authors:** Antonia L Newman, Thomas D Meyer

**Affiliations:** Northumberland, Tyne and Wear NHS Foundation Trust, St Nicholas Hospital, Gosforth, Newcastle upon Tyne, NE3 3XT UK; Institute of Neuroscience, Newcastle University, Ridley Building, Newcastle upon Tyne, NE1 7RU UK

**Keywords:** Bipolar disorder, Euthymia, Impulsivity, Self-report, Delay of gratification, Response inhibition

## Abstract

Because impulsivity is part of the presentation of bipolar disorder (BD) and is associated with its course, this systematic review presents the evidence whether increased impulsivity is present in a stable, euthymic mood and therefore potentially a vulnerability marker for BD. A multi-faceted model of impulsivity was adopted to explore how different facets may relate differently to BD. The evidence was explored in relation to studies employing measures of trait impulsivity (in self-report format) and studies exploring impulsivity with behavioural paradigms. Behavioural paradigms were separated into studies measuring response inhibition and those measuring the ability to delay gratification. Twenty-three papers met the inclusion criteria. Most studies using self-report measures found significant differences between euthymic BD patients and healthy controls. There was little evidence of increased impulsivity as measured by behavioural paradigms. Most studies found no significant difference in response inhibition between groups, though it is possible that much of the literature in this area was underpowered to detect an effect. Only five studies explored delay of gratification, of which the two methodologically strongest studies found no group differences. In conclusion, there is evidence that euthymic patients with BD report increased impulsivity when using self-ratings. However, there is currently limited evidence of impulsivity on behavioural measures assessing response inhibition, and this might be restricted to more severe cases. More research is needed on the ability to delay gratification before drawing any conclusions. However, to establish facets of impulsivity as vulnerability markers, future studies should include at-risk individuals to evaluate whether self-rated or behavioural impulsivity precedes the onset of BD.

## Review

### Introduction

Impulsivity is one of the DSM diagnostic criteria for mania, listing as ‘excessive involvement in pleasurable activities that have a high potential for painful consequences’ (American Psychiatric Association 2013). Furthermore, impulsivity in bipolar disorder (BD) has been linked to poorer outcome, including a more severe course and suicidality (Watkins and Meyer 2013; Swann et al. 2009a). As a result, there has been a lot of interest in the relationship between impulsivity and BD.

Several theoretical models about BD include elements relating to impulsivity. The cognitive-behavioural model of Mansell et al. (2007) describes impulsive behaviours in relation to ‘ascent behaviours’ which escalate an individual towards mania. Another model proposes that the Behavioural Activation System (BAS; Gray 1994) as a psychobiological system underlying motivation and approach behaviour is related to BD (Alloy and Abramson 2010). For example, Alloy et al. (2012) propose that the onset of mania is related to BAS dysregulation, leading to extreme reward-seeking behaviour. Extreme reward seeking is one facet that is linked to impulsivity, as individuals may pursue reward whilst disregarding consequences.

Impulsivity is however a broad concept, and the broad nature of impulsivity is well captured in the definition by Daruna and Barnes (1993, p. 23) who stated that ‘impulsivity encompasses a range of actions which are poorly conceived, prematurely expressed, unduly risky or inappropriate to the situation and that often result in undesirable consequences’. Researchers agree that impulsivity is multi-faceted (Barratt 1994; Reynolds et al. 2006). However, there is little consensus on the constructs referred to under the umbrella term ‘impulsivity’. For example, Whiteside and Lynam (2001) factor-analysed several widely used self-report impulsivity measures resulting in their four-factor model of impulsivity: ‘lack of premeditation’, ‘lack of perseverance’, ‘sensation seeking’ and ‘urgency’. The new factor here is urgency meaning the tendency to act impulsively in an emotional state, be it positive or negative (Cyders et al. 2007). Barratt et al. (Patton et al. 1995; Stanford and Barratt 1992) considered self-ratings, behavioural tasks and animal research resulting in a three-factor model of impulsivity: ‘attentional’, ‘motor’ and ‘non-planning’ impulsivity, leading to the development of the Barrett Impulsivity Scale (BIS). There is therefore no accepted consensus on the components comprising impulsivity as a trait measured by self-reports.

With regards to behavioural manifestations of impulsivity, two facets can be repeatedly recognised. They can be broadly categorised as follows: (a) lack of response inhibition (RI) defined by Verbruggen and Logan (2008, p. 418) as ‘the suppression of no-longer required or inappropriate actions’; (b) inability to delay gratification defined by Arce and Santisteban (2006, p. 214) as ‘the inability to weigh the consequences of immediate and future events and, consequently, delay gratification’. These behavioural manifestations of impulsivity (or versions closely mapping on to them) have been identified by numerous researchers with potential links to separate brain systems (e.g. Winstanley et al. 2006; Chudasama et al. 2003; Mobini et al. 2002; Swann et al. 2002; Brunner and Hen 1997). One additional fact to keep in mind is that the different facets of impulsivity are also often not strongly correlated. This is especially true for the association between self-report and behavioural measures (e.g. Reynolds et al. 2006) and between different behavioural measures (e.g. Lane et al. 2003). Classifying an individual as ‘impulsive’ can therefore have different meanings depending on the facets measured.

Research with acutely manic patients suggests they score higher on self-report impulsivity measures (Strakowski et al. 2009; Swann et al. 2003) and are more impulsive on tasks measuring RI (Najt et al. 2005; Strakowski et al. 2009) and ability to delay gratification (Clark et al. 2001; Strakowski et al. 2009). Impulsivity may not, however, be confined to the manic phase of the illness. For example, Swann et al. (2009b) found a sample of BD in different mood states that was high in impulsivity with respect to self-ratings and delay discounting tasks. Murphy et al. (2001) found deficits in the delay of gratification in depressed bipolar patients. However, it is not clear yet whether impulsivity is only a symptom of BD or a trait of individuals experiencing BD, and this is important for a better understanding of the aetiology of BD and for adapting psychological treatments.

To explore whether impulsivity is a trait of those with BD, research over the last decade has explored whether BD patients show elevated impulsivity during euthymic mood. A former narrative review on impulsivity in BD (Najt et al. 2007) suggested that there were both state and trait components to impulsivity. However, this was based upon only a small number of studies, reflecting the limited research available at that time. Additionally, some behavioural paradigms (e.g. Continuous Performance Test (CPT), Fleck et al. 2005) do not focus on impulsivity but provide indices related to impulsivity, which have not always been covered in such reviews. Therefore, to fully evaluate the current research on impulsivity in euthymia, it is important to consider studies using such paradigms, though impulsivity may not be the focus of the research.

The aim of this systematic review is therefore to explore whether there is evidence for increased impulsivity in the euthymic stage of BD if one looks at both self-reported impulsivity and two commonly identified behavioural manifestations of impulsivity: response inhibition and ability to delay gratification.

## Method

A systematic search of the literature was undertaken. Included papers met the following criteria: (a) adult BD patients with diagnosis confirmed through structured clinical interview; (b) BD patients are described as euthymic, inter-episode or in remission, and some measure of current mood must have been used; (c) the study measured a form of impulsivity; as many tasks could potentially be considered measures of impulsivity, setting limits for inclusion in the review was necessary (we included all paradigms that have previously been used in published BD studies to explicitly investigate impulsivity, even if used in the study to explore a different concept); and (d) studies that included a healthy control group. Exclusion criteria were as follows: (a) if the impulsivity measure used was the CPT, but the study failed to report the ‘false alarm’/‘commission error’ rate; (b) tests conducted with prior experimental manipulation (e.g. mood induction); (c) qualitative studies; and (d) multiple publications reporting the same study, including only the most relevant paper.

Variants of the key terms ‘impulsivity’, ‘bipolar disorder’ and ‘euthymia’ were searched in the databases PsycINFO, Medline, Web of Knowledge and Scopus in November 2012. Depending on the database, explode functions, wild cards and speech marks were used, and combined searches using the Boolean terms OR and AND. For impulsivity, concepts such as ‘response inhibition’ were also entered (full list available from the authors). No date range limits were set. The concept of euthymia impacted upon search sensitivity and was removed from the search.

**Figure 1:**
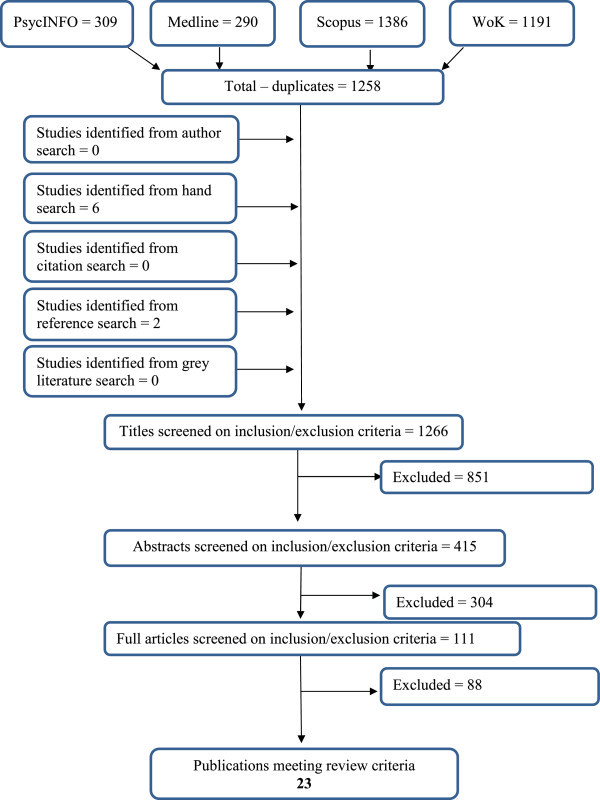
**Number of papers identified during the search and the selection process.**

The journals *Bipolar Disorder* and *Journal of Affective Disorders* were hand-searched to identify further articles. All these were studies employing the CPT, but as a measure of attention rather than impulsivity. The electronic search was therefore expanded to search for the term ‘Continuous Performance Test’ to ensure that relevant papers were identified. Key authors were searched within the databases and contacted to identify unpublished studies. References of the included studies were searched. A citation search was also conducted in PsycINFO and Scopus. Figure 1 illustrates the number of papers identified during the search and the selection process. To assess methodological rigour, the papers were evaluated against a checklist of key issues exploring validity. An evaluation grid was designed and tailored to the review question, adapted from others (e.g. Hadorn et al. 1996; Scottish Intercollegiate Guidelines Network 2004). This evaluation grid was used as a guide to evaluate the quality of each paper (available from the authors on request).

## Results

The main characteristics of the studies included are outlined in Table 1. Studies employing self-report and behavioural measures are discussed separately. Behavioural measures are subdivided into those exploring response inhibition (RI) and those exploring ability to delay gratification. The detailed studies' performance against the evaluation checklist can be obtained from the authors. Key criteria included, for example whether patients were clearly defined as euthymic and controls, were verified as having no history of psychiatric illness. These issues are given particular importance in the methodological evaluation. Effect sizes were estimated and are reported in the respective tables. The effect sizes were calculated using the formula:


as recommended by Thalheimer and Cook (2002).

**Table 1:** **Summary of studies included in the review**

Author and year of publication	Main focus of paper	Relevant aims or hypotheses	Participants	Clinical measures	Impulsivity measure/s	Statistical analysis	Main results relevant to impulsivity
Ancin et al. (2010)	Sustained attention	No aims or hypotheses relevant to this review	143 Euthymic BD patients	SCID	Computerised degraded stimulus CPT	*T* test and ANOVA	BD group had longer reaction times than controls. No group difference in false alarm rate or response criterion score in any of three CPT blocks
			101 Healthy controls	HDRS			
				YMRS		Median test for non-parametric data	
				Vocabulary subtest of WAIS			
Bora et al. (2007)	Cognitive impairment	No aims or hypotheses relevant to this review	65 Euthymic BD-I patients (40 euthymic psychotic, 25 euthymic non-psychotic)	SCID	Conners' CPT II	MANOVA	Previously psychotic euthymic BD patients made more commission errors than controls. No difference between non-psychotic euthymic BD patients and controls on commission errors. No group differences in hit reaction time
				YMRS			
			30 Healthy controls	HDRS			
				Brief Psychiatric Rating Scale			
Brooks et al. (2010)	Sustained attention	No aims or hypotheses relevant to this review	16 Euthymic BD patients over age 50	MINI	Conners' CPT II	Mann-Whitney *U* test	No group differences in commission error rate or hit reaction time
			11 Healthy controls	MADRS			
				YMRS			
Ekinci et al. (2011)	Impulsivity	Hypothesis: ‘some clinical appearances would be differentially related to impulsivity in subjects with BD’	71 Euthymic BD-I patients	SCIDI and II	BIS-11	Pearson’s correlation and ANOVA	Patient’s scores were significantly higher on total BIS score and on all subscales. They also scored more highly on the impulsiveness scale of the TCI
			50 Healthy controls	YMRS	Impulsiveness scale of Temperament and Character Inventory (TCI)		
				HDRS			
Etain et al. (2013)	Impulsivity	Aim: ‘to study trait-impulsiveness in a large population of euthymic BD patients and healthy subjects’	385 Euthymic BD patients	MADRS	BIS-10	Wilcoxon and Mann-Whitney *U* test	Patients’ scores were significantly higher than controls on BIS total and all subscale scores
			185 Healthy controls	BRMAS			
				Diagnostic Interview of Genetic Studies		Kruskal-Wallis	
Fleck et al. (2005)	Sustained attention	No aims or hypotheses relevant to this review	25 Manic and mixed BD-I patients with psychotic features	SCID	Computerised degraded-stimulus CPT	ANOVA	Patients did not differ to controls on response bias (beta) outcome of CPT
				YMRS			
			23 Remitted BD-I patients	HDRS			
			28 Healthy controls	Scale for the Assessment of Positive Symptoms			Patients had significantly slower reaction times than controls
Henna et al. (2013)	Impulsivity	Main hypothesis: ‘euthymic BD and unipolar subjects have higher impulsivity than unaffected relatives and healthy controls’	54 Euthymic BD patients	SCID	BIS 11A	ANOVA	Patients scored more highly than unaffected relatives and healthy controls on BIS total, motor and non-planning subscales
			136 Healthy controls	YMRS			
			14 Unaffected relatives	HDRS			
			25 Euthymic unipolar patients				
							Patients scored higher than controls on attentional impulsivity subscale
Ibanez et al. (2012)	Decision-making and reward processing	No aims or hypotheses relevant to this review	13 Euthymic BD-II patients	SCID	Iowa Gambling Task	ANOVA	Only one significant difference between BD group and controls on outcomes of Iowa Gambling Task. BD patients were impaired compared to controls on blocks 4 and 5 of the task
			12 ADHD patients	MADRS	BIS		
			25 Healthy controls	YMRS	Go/no go task		
				BDI			
				State-Trait Anxiety Inventory			
				Rey Auditory Verbal Learning Test			
Iosifescu et al. (2009)	Cognitive function	No aims or hypotheses relevant to this review	20 Remitted BD-I and BD-II patients	HDRS	Conners' CPT	*T* tests	BD patients made significantly more commission errors than controls
				YMRS			
			10 Healthy controls	Affective Disorder Evaluation			
Kaladjian et al. (2009)	Response inhibition	No aims or hypotheses relevant to this review	27 Euthymic BD-I patients	SCID	Go/no go task	*T* tests	No group differences on impulsivity outcomes, including response bias (beta) and reaction time
			25 Healthy controls	YMRS			
				HDRS			
				NART			
Kolur et al. (2006)	Sustained attention	No aims of hypotheses relevant to this review	30 Euthymic BD patients ages 17 to 30. Illness duration <5 years and no more than two affective episodes	YMRS	CPT	Wilcoxon signed rank test	No group differences on commission errors. Patients had significantly slower reaction time than controls
				HDRS			
				MMSE		Mann-Whitney *U* test for subgroup analyses	Within BD group, patients with a history of two mood episodes made significantly more commission errors than those with only one previous episode
			30 Healthy controls	MINI			
Kung et al. (2010)	Sustained attention	No aims of hypotheses relevant to this review	51 Euthymic BD patients (22 BD-I and 29 BD-II)	HDRS	Conners' CPT-II	Pearson’s correlation	BD-I patients had significantly longer reaction times and more commission errors than BD-II patients and healthy controls
			20 Healthy controls	YMRS		MANOVA	
Lewis et al. (2009)	Impulsivity	Aim: ‘to examine the relationship of impulsivity to clinical status and personality characteristics in patients with BD’	36 Remitted BD patients	Clinical Global Impressions Scale	BIS-11	ANCOVA	No difference between remitted BD patients and controls on BIS total scores or any of the subscales
			25 Subsyndromal BD patients	MADRS		Pearson’s correlation	
			45 Syndromal BD patients	YMRS			
			30 Healthy controls	SCID			
Lombardo et al. (2012)	Impulsivity	Hypothesis: ‘euthymic individuals with BD and their clinically unaffected siblings would have higher levels of trait impulsivity compared to healthy subjects’	54 Euthymic BD-I patients	SCID	BIS-11	Linear mixed model	Patients had significantly elevated BIS total and subscale scores compared to siblings and healthy controls
			57 Clinically unaffected siblings	GAF			
				HDRS			
			49 Healthy controls	YMRS			
Malloy-Diniz et al. (2011)	Impulsivity	Aim: ‘to assess different impulsivity components in BD sub-grouped by suicidal attempt and healthy controls’	95 Euthymic BD patients (41 with lifetime history of suicide attempt)	MINI	CPT-II	Mann-Whitney	BD patients made more commission errors than controls on the CPT. They had slower hit reaction times than the controls
				Brazilian version of BDI	Iowa Gambling Task		
			94 Healthy controls	YMRS			
				Raven’s progressive matrices			
							BD patients were impaired compared to controls on blocks 3,4 and 5 and overall task performance of the Iowa Gambling Task
Martino et al. (2008)	Cognitive functioning	No aims of hypotheses relevant to this review	20 Euthymic BD older adults	YMRS	CPT	*T* test	No difference between groups on any of the outcome measures of the CPT
			20 Age-matched healthy controls	HDRS			
				Mini-mental state examination			
				Unified Parkinson's Disease Rating Scale-III			
				GAF			
				SCID			
				WAIS			
Martino et al. (2011)	Decision making	Aim: ‘to compare a large population of patients with BD types I and II strictly defined as euthymic with healthy controls on measures of decision making’	85 Euthymic BD patients	SCID	Iowa Gambling Task	ANOVA	No difference between BD-I or BD-II patients and controls on any of IOWA outcome measures
			34 Healthy controls	HDRS			
				YMRS			
Peluso et al. (2007)	Impulsivity	Hypothesis: ‘bipolar subjects would have higher levels of trait impulsivity than the comparison group’	24 Depressed bipolar patients	HDRS	BIS	ANCOVA	Controls had significantly lower scores on all BIS scales compared to euthymic BD patients
			24 Depressed unipolar patients	SCID			
			12 Euthymic bipolar patients				
			10 Euthymic unipolar patients				
			51 Healthy controls				
Strakowski et al. (2010)	Impulsivity	Aim: ‘to determine whether abnormalities of impulse control persist across the course of BD’	31 Euthymic BD patients	SCID	Logan stop signal task	ANCOVA	Euthymic BD patients did not differ from controls on any of the behavioural tasks
			48 Healthy controls	YMRS	Delayed reward task		
			26 Depressed BD patients	MADRS	Degraded stimulus CPT		BIS total score, motor subscale and non-planning subscale were elevated in BD patients compared to controls. ttentional subscale did not differ to controls
				NART			
Swann et al. (2003)	Impulsivity	Aims: to investigate impulsivity in manic episodes of BD, compared to euthymic BD patients and controls	25 Euthymic BD patients	SCID	BIS	ANOVA	BIS total and sub-scale scores were elevated in euthymic BD patients compared to controls
			14 Manic BD patients	SADS-C	IMT-DMT version of CPT		
			35 Healthy controls				
							No difference between euthymic BD patients and controls on IMT-DMT task
Swann et al. (2004)	Impulsivity	Hypotheses: ‘impulsivity as a trait (BIS-11) would be elevated in either substance abuse or in inter-episode BD, and would be elevated more in subjects with BD and substance abuse’	30 Inter-episode BD patients (12 with SA history)	SCID	BIS-11	ANOVA	BD patients showed elevated BIS total and subscale scores compared to controls patients
				SADS-C	IMT-DMT version of CPT		
			35 Individuals with history of SA				
			37 Healthy controls				
							No difference in commission errors between BD patients and controls on IMT-DMT task
Thompson et al. (2009)	Executive control	No aims or hypotheses relevant to this review	63 Euthymic BD patients	SCID	Vigil CPT	*T* tests	No group difference in commission error rates
			63 Healthy controls	YMRS		ANOVA	
				HDRS			
				BDI			
				Altman Mania Rating Scale			
				NART			
				MMSE			
Yechiam et al. (2008)	Decision making	No aims relevant to this review	14 Remitted BD patients	SCID	Iowa Gambling Task	ANOVA	No group differences on outcomes for Iowa Gambling Task
			14 Acute BD patients	YMRS			
			25 Healthy controls				

BDI, Beck Depression Inventory; BIS, Barratt Impulsivity Scale; BRMAS, Bech Rafaelsen Mania Scale; CPT, Continuous Performance Test; HDRS, Hamilton Depression Rating Scale; MADRS, Montgomery-Asberg Depression Rating Scale; MINI, Mini-International Neuropsychiatric Interview; MMSE, Mini Mental State Examination; NART, National Adult Reading Test; SADS-C; Schedule for Affective Disorder and Schizophrenia- Change version; SCID, Structured Clinical Interview for DSM-IV; WAIS, Weschler Adult Intelligence Scale; YMRS, Young Mania Rating Scale.

### Self-report personality measures

Ten of the studies used self-report tools measuring trait impulsivity (see Table 2). All used the BIS (Patton et al. 1995) which is considered as a reliable and valid tool. The BIS provides a total score as well as subscale scores of motor impulsivity (the tendency to act without thinking), attentional impulsivity (difficulties to sustain attention) and non-planning impulsivity (acting without considering the future). Stanford et al. (2009) suggest that individuals can be considered as highly impulsive with a total BIS score over 72. The study by Henna et al. (2013) employed version 11A of the BIS. This version was an early working version of the BIS-11, which the authors advise against using, thus this paper did not score highly on reliability of the measures employed.

**Table 2:** **BIS total score: effect sizes**

Study	BD group	Control group	Effect size
	Mean (SD)	Mean (SD)	
Ekinci et al. (2011)	*N* = 71	*N* = 50	3.97
	74.33 (7.85)	50.36 (3.48)	
Etain et al. (2013)^a^	*N* = 385	*N* = 185	0.65
	66.1 (11.1)	59.5 (8.4)	
Henna et al. (2013)	*N* = 54	*N* = 136	2.02
	73.9 (13.2)	53.2 (9.1)	
Ibanez et al. (2012)	*N* = 13	*N* = 25	0.83
	54.2 (22.3)	40.9 (12.8)	
Lewis et al. (2009)	*N* = 36	*N* = 30	-0.23
	58.7 (8.2)	60.8 (10.0)	
Lombardo et al. (2012)	*N* = 54	*N* = 49	2.45
	72.9 (12.1)	52.4 (8.9)	
Peluso et al. (2007)	*N* = 12	*N* = 51	1.99
	75.0 (15.1)	56.1 (8.2)	
Strakowski et al. (2010)	*N* = 28	*N* = 35	1.07
	61 (11)	51 (8)	
Swann et al. (2003)	*N* = 22	*N* = 35	1.43
	77.1 (13.8)	59.9 (9.3)	
Swann et al. (2004)	Not provided	Not provided	

^a^Means obtained from author as not reported in original paper.

Methodologically strong papers using self-report impulsivity measures include those by Ekinci et al. (2011) and Strakowski et al. (2010). The paper by Swann et al. (2003) did not verify the BD group as euthymic through interview or using symptom measure cut-offs. It is also not totally clear whether the control group were assessed for lifetime history of psychiatric symptoms. The criteria for euthymia adopted by Peluso et al. (2007) and Swann et al. (2004) were less strict, and the sample sizes were on the lower end. Ibanez et al. (2012) also had a small sample sizes, running a risk of being underpowered.

Of the ten studies in this section, all but two (Lewis et al. 2009; Ibanez et al. 2012) found significant differences between euthymic bipolar patients and controls on BIS total score. The effect size calculations were completed and all but one of these studies found large effects (*d* = 1.07 to 3.96). Five of the seven studies reporting mean scores for the groups found mean BIS scores for the euthymic group of over 72, which is suggested by Stanford et al. (2009) as highly impulsive.

The Ibanez et al. (2012) study was probably underpowered because the estimated effect size for their group comparison was large (*d* = 0.83). Nevertheless, it seems noteworthy that the mean BIS score of their BD group was at the lower end of ‘normal’ range and the control group's mean was 40, which is very low, indicating either being over-controlled or potentially having completed the questionnaire incorrectly (Stanford et al. 2009).

Of the nine studies reporting subscale scores (see Tables 3, 4 and 5), eight found significantly higher scores for the BD group on the subscales of non-planning and motor impulsivity. Seven of these studies also found differences on the ‘attentional impulsivity’ subscale. Mostly, these results represented large effect sizes.

**Table 3:** **BIS motor impulsivity score: effect sizes**

Study	BD group	Control group	Effect size
	Mean (SD)	Mean (SD)	
Ekinci et al. (2011)	*N* = 71	*N* = 50	2.73
	24.90 (3.23)	17.02 (2.25)	
Etain et al. (2013)^a^	*N* = 385	*N* = 185	0.41
	23.0 (4.57)	21.29 (3.34)	
Henna et al. (2013)	*N* = 54	*N* = 136	1.71
	26.1 (5.0)	19.4 (3.5)	
Lewis et al. (2009)	*N* = 36	*N* = 30	-0.69
	20.2 (3.3)	22.8 (4.3)	
Lombardo et al. (2012)	*N* = 54	*N* = 49	1.56
	26.1 (4.9)	19.8 (3.1)	
Peluso et al. (2007)	*N* = 12	*N* = 51	1.33
	24.3 (6.7)	18.6 (3.7)	
Strakowski et al. (2010)	*N* = 28	*N* = 35	1.03
	23 (5)	19 (3)	
Swann et al. (2003)	*N* = 22	*N* = 35	1.14
	27.7 (4.8)	22.8 (4.0)	
Swann et al. (2004)	*N* = 15	*N* = 37	0.23
	23.5(3.9)	22.6 (4.0)	

^a^Means obtained from author as not reported in original paper.

**Table 4:** **BIS non-planning impulsivity score: effect sizes**

Study	BD group	Control group	Effect size
	Mean (SD)	Mean (SD)	
Ekinci et al. (2011)	*N* = 71	*N* = 50	2.38
	28.11 (2.86)	21.39 (2.70)	
Etain et al. (2013)^a^	*N* = 385	*N* = 185	0.52
	26.0 (5.1)	23.5 (4.1)	
Henna et al. (2013)	*N* = 54	*N* = 136	1.49
	26.6 (6.0)	19.7 (4.1)	
Lewis et al. (2009)	*N* = 36	*N* = 30	0.08
	23.9 (4.4)	23.5 (5.2)	
Lombardo et al. (2012)	*N* = 54	*N* = 49	1.55
	28.0 (5.5.)	20.3 (4.4)	
Peluso et al. (2007)	*N* = 12	*N* = 51	2.00
	31.2 (6.8)	22.6 (3.7)	
Strakowski et al. (2010)	*N* = 28	*N* = 35	0.90
	24 (5)	20 (4)	
Swann et al. (2003)	*N* = 22	*N* = 35	1.24
	29.0 (6.2)	22.4 (4.8)	
Swann et al. (2004)	*N* = 15	*N* = 37	0.83
	26.8 (5.8)	22.6 (4.7)	

^a^Means obtained from author as not reported in original paper.

**Table 5:** **BIS attentional impulsivity score: effect sizes**

Study	BD group	Control group	Effect size
	Mean (SD)	Mean (SD)	
Ekinci et al. (2011)	*N* = 71	*N* = 50	0.98
	21.20 (3.94)	18.30 (1.60)	
Etain et al. (2013)^a^	*N* = 385	*N* = 185	0.61
	17.1 (4.2)	14.7 (3.3)	
Henna et al. (2013)	*N* = 54	*N* = 136	1.72
	21.1 (4.7)	14.0 (3.9)	
Lewis et al. (2009)	*N* = 36	*N* = 30	0.03
	14.6 (3.1)	14.5 (2.8)	
Lombardo et al. (2012)	*N* = 54	*N* = 49	1.03
	18.7 (4.2)	12.3 (3.4)	
Peluso et al. (2007)	*N* = 12	*N* = 51	1.41
	19.6 (4.7)	14.9 (3.0)	
Strakowski et al. (2010)	*N* = 28	*N* = 35	0.69
	14(4)	12 (2)	
Swann et al. (2003)	*N* = 22	*N* = 35	1.47
	20.7 (4.7)	14.8 (3.6)	
Swann et al. (2004)	*N* = 15	*N* = 37	0.97
	18.5 (4.3)	15.1 (3.2)	

^a^Means obtained from author as not reported in original paper.

The study by Strakowski et al. (2010) is the only longitudinal study we identified. They initially assessed manic and mixed BD-I patients on a range of impulsivity measures. They then followed up participants after 1 year. They found that after becoming euthymic, patients' BIS total, non-planning and motor subscales remained elevated compared to controls and did not differ significantly from scores when manic. The ‘attentional subscale’ reduced slightly so that it no longer significantly differed to controls, although effect size calculations still indicated a medium effect size (*d* = 0.69).

The methodological strength of the study by Strakowski et al. (2010) and the general consensus among studies provide strong evidence that impulsivity as measured by self-reports remains higher in euthymia than for healthy controls and may be a trait of those who experience BD. However, Lewis et al. (2009) found no differences on BIS scores. They had adopted a very strict definition of euthymia. However, four out of the seven papers finding significant differences in self-reported impulsivity also had strict definitions of euthymia, making this explanation unlikely. Another possibility highlighted by Lewis et al. is that they might have been recruited from a setting with less complex or severe cases than the recruitment sites in other studies. Therefore, it may be that the severity of illness during the acute stage is related to the level of impulsivity during euthymia. The study of Ekinci et al. (2011) was the only one to use another self-report measure, the Impulsiveness scale of the novelty-seeking dimension in the Temperament and Character Inventory (TCI; Cloninger et al. 1993). The TCI is a 240-item personality questionnaire. Novelty seeking is considered a ‘higher order’ temperament within the questionnaire and consists of four ‘lower order’ traits, of which impulsiveness is one. Ekinci et al. found significant differences between euthymic BD patients and controls on this scale representing a very large effect size (*d =* 3.06).

Overall, there were substantially more BD-I than BD-II patients included in these studies. Strakowski et al. (2010), Lombardo et al. (2012) and Ekinci et al. (2011) used exclusively BD-I patients, whilst 75% of the participants in the study of Etain et al. (2013) and 90% in the study of Lewis et al. (2009) had been diagnosed with BD-I. The remaining studies did not report the type of BD diagnosis. The samples represented here may therefore be more representative of individuals who experience full manic episodes and are diagnosed with BD-I. In summary, there is substantial evidence that euthymic BD patients self-report higher levels of impulsivity than healthy controls. This is particularly the case for facets labelled as ‘motor impulsivity’ and ‘non-planning impulsivity’, though six of the eight studies also found higher levels of attentional impulsivity.

### Behavioural measures of impulsivity

#### Response inhibition measures

Fifteen studies used tasks identified as measuring response inhibition (RI) using the go/no go task, versions of the CPT and a stop-signal task. Of these, only five explicitly examined impulsivity. The others mostly focused on sustained attention using a version of the CPT, from which the commission errors (false alarms), hit reaction time (HRT) and response criterion (beta) can be considered as measures of impulsive behaviour.

Most of these studies were good with respect to group allocation and had clear inclusion/exclusion criteria. Using the grid, the reliability of the impulsivity measures used was not rated as highly as for the self-report studies. This is mainly due to the fact that reliability for the measures used (mainly variations of the CPT) is less often formally established since the behavioural indices (e.g. error rates) are known to be often influenced by factors such as tiredness, practice or time of day. The unknown or low reliability of these measures is not necessarily a problem but needs to be kept in mind when drawing conclusions.

As described in the self-report section, the Strakowski et al. (2010) paper is methodologically strong and included multiple measures of impulsivity. The study of Bora et al. (2007) was also strong, with a strict definition of euthymia and clinical symptoms assessed by a trained clinician. The paper by Swann et al. (2003) included a measure of RI. The Brooks et al. (2010) study did not report how euthymia was defined and had a small sample size. Lastly, the Malloy-Diniz et al. (2011) study used relatively high cut-offs on symptom measures to define euthymia. This paper is therefore likely to contain subsyndromal patients.

*Continuous Performance Test (CPT).* For the CPT, commission errors are the main outcome linked to impulsivity. Eleven papers reported commission error rates, of which four found significant differences between euthymic BD patients (or a subset of BD patients) and controls (see Table 6).

**Table 6:** **Continuous Performance Test: effect sizes**

Study	BD group	Control group	Effect size
	Mean (SD)	Mean (SD)	
Ancin et al. (2010)	*N* = 141	*N* = 101	
False alarm rate, block 3	10.6 (12.3)	8.6 (11.6)	0.167
Response criterion, block 3	0.65 (0.72)	0.53 (0.87)	0.15
Brooks et al. (2010)	*N* = 16	*N* = 11	
Commission errors	15.5 (7.9)	10.5 (5.5)	0.68
Fleck et al. (2005)	*N* = 23	*N* = 28	
Response criterion	0.49 (0.22)	0.52 (0.20)	-0.14
Iosifescu et al. (2009)	*N* = 20	*N* = 10	
Commission errors	12.41 (4.82)	7.50 (4.93)	1.01
Kolur et al. (2006)	*N* = 30	*N* = 30	
Commission error	21.40 (29.91)	12.73 (7.15)	0.47
Malloy-Diniz et al. (2011)	*N* = 95	*N* = 94	
Commission errors	16.17 (8.76)	10.26 (7.2)	0.74
Martino et al. (2008)	*N* = 20	*N* = 20	
False alarm rate	6.7 (6.1)	4.0 (3.4)	0.57
Strakowski et al. (2010)	*N* = 31	*N* = 35	
Response criterion	0.69 (0.28)	0.67 (0.30)	0.07
Swann et al. (2003)	*N* = 25	*N* = 35	
DMT commission errors	21.1 (18.8)	17.9 (15.5)	0.19
Swann et al. (2004)	*N* = 37	*N* = 16	
DMT commission errors	13.6 (9.0)	18.4 (15.6)	-0.44
Thompson et al. (2009)	*N* = 63	*N* = 63	
Commission errors	2.61 (3.10)	1.73 (2.23)	0.33

Only two papers found their sample of euthymic BD group as a whole to make more commission errors than controls. Both papers showed medium-large effect sizes for the group differences. One of these studies (Malloy-Diniz et al. 2011) included a sample that was not clearly euthymic, and the results may represent higher commission errors associated with residual symptoms. The second paper was by Iosifescu et al. (2009). They specifically included BD participants reporting cognitive deficits and compared them to healthy controls. They had methodological reasons for this, as they undertook a medication trial for improving cognitive symptoms. However, their sample was therefore not representative of the general BD population.

Bora et al. (2007) separated those with and without a history of psychotic symptoms. Those with a history of psychotic symptoms made significantly more commission errors than both controls and non-psychotic patients. Kung et al. (2010) found that BD-I patients made more commission errors than controls but not BD-II patients. Neither Kung et al. nor Bora et al. reported the BD data for the whole group.

The perusal of effect sizes suggests that some studies might have been underpowered to detect significant group differences. The studies by Brooks et al. (2010), Martino et al. (2008) and Kolur et al. (2006) all showed medium effect sizes (*d =* 0.47 to 0.68) but failed to find significant group differences. These studies had relatively small samples. Thompson et al. (2009) had larger groups, but found small-medium effects, so still may have been underpowered. However, Ancin et al. (2010) included a large sample but only found trivial effects.

Three studies (Ancin et al. 2010; Fleck et al. 2005; Strakowski et al. 2010) reported beta - a response criterion measure indicating liberal versus conservative responding to target stimuli. Beta can also be considered as a measure of impulsivity, but no study found significant group differences, and the effect sizes were trivial. The last CPT outcome linked to impulsivity is HRT. Of the nine studies reporting HRT, four found that euthymic BD patients were slower than healthy controls. Indeed, all studies that found BD patients were slower to respond than controls, though not all found a significant difference. Ordinarily, *faster* RTs would be associated with impulsivity. A number of studies finding slower reaction times postulate that it is possible that euthymic patients sacrifice speed for accuracy.

*Go/no go task*. Two studies (Kaladjian et al. 2009; Ibanez et al. 2012) employed the go/no go task (see Table 7). Participants must press a button when one type of stimulus appears and withhold responding when a different stimulus is presented. Commission errors are the outcome indicating impulsivity. Neither study found significant group differences. However, while the Ibanez et al. study found a large effect size for difference in commission error rate (*d* = 0.89), the effect size was trivial in the Kaladjian et al. study, and the BD group mean commission error rate was lower than the control group.

**Table 7:** **Go/no go response inhibition: effect sizes**

Study	BD group	Control group	Effect size
	Mean (SD)	Mean (SD)	
Ibanez et al. (2012)	*N* = 13	*N* = 25	
Commission errors	7.6 (19.8)	0.37 (2.0)	0.89
Kaladjian et al. (2009)	*N* = 20	*N* = 20	
Commission errors	7.3 (5.5)	8.9 (7.2)	-0.22

In summary, the majority of studies looking at RI failed to detect significant differences between euthymic BD patients and controls. The four studies reporting significant effects had either pre-selected or subgroups of BD patients (e.g. those with cognitive deficits). Therefore, there is limited evidence that euthymic BD patients perform more impulsively on RI measures than controls. However, there is tentative evidence that euthymic BD patients make more errors of commission in some tasks than controls but that some of the research has been underpowered to detect such an effect.

### Delay of gratification measures

Five papers used paradigms measuring the ability to delay gratification (see Table 8). Two were explicitly exploring impulsivity. The others explored decision-making more generally. However, the outcomes of the paradigms used in the papers all have relevance to this facet of impulsivity.

**Table 8:** **Delay of gratification task: effect sizes**

Study	BD group	Control group	Effect size
	Mean (SD)	Mean (SD)	
Ibanez et al. (2012)	*N* = 13	*N* = 25	
IGT net score	1526.5 (483.0)	1847.1 (564.1)	-0.60
IGT blocks 1 and 2	-1.3 (7.9)	0.65 (7.1)	-0.26
IGT blocks 3 and 4	1.0 (8.4)	4.3 (8.2)	-0.41
Malloy-Diniz et al. (2011)	*N* = 95	*N* = 94	
IGT net score	3.89 (24.28)	20.57 (23.61)	-0.70
IGT block 1	1.87 (4.47)	1.2 (5.99)	0.13
IGT block 2	0.49 (5.18)	2.14 (6.80)	-0.28
IGT block 3	1.20 (6.72)	5.68 (6.84)	-0.66
IGT block 4	1.40 (7.68)	6.93 (8.07)	-0.70
IGT block 5	1.52 (8.92)	6.78 (9.13)	-0.58
Strakowski et al. (2010)	*N* = 31	*N* = 35	
Delayed reward task (% impulsive)	31 (26)	28 (23)	0.25

The methodologically strong studies in this area were Strakowski et al. (2010) and Martino et al. (2011). Yechiam et al. (2008) however did not specify clear criteria for defining euthymia, and 36% of their ‘remitted’ group was still experiencing residual psychotic symptoms. They also did not measure or report the severity of mood symptoms in the remitted group. The sample is therefore likely to have included subsyndromal patients. The methodological assessment of the Malloy-Diniz et al. (2011) and Ibanez et al. (2012) had already been described before.

Four out of the five studies used the *Iowa Gambling Task* (IGT; Bechara et al. 1994). Participants are asked to choose a card from four available decks and win or lose money depending on their choice. Decks A and B provide high rewards but occasional high loses, leading to a net loss over time. Decks C and D have smaller rewards but less loses, resulting overtime in overall profit. Impulsive participants are expected to be more likely to draw from decks A and B, preferring larger immediate rewards. Of the studies using the IGT, two (Martino et al. 2011; Yechiam et al. 2008) found no significant group differences. Malloy-Diniz et al. (2011) however found BD patients are impaired to controls on the overall task performance (measured by net task score) and on blocks 3, 4 and 5 of the task. Only in *post hoc* analyses Ibanez et al. (2012) found a significant difference between euthymic patients and controls on blocks 3 and 4 (combined). Group sizes in this study were however small (only 13 patients in BD group). The effect size calculations indicate medium-large effects on net IGT score (*d =* 0.60), suggesting that the study may not have been sufficiently powered to detect an effect.

Strakowski et al. (2010) administered a delayed reward task to assess the ability to delay gratification. Participants use a computer mouse to select the letter A or B on the screen. Selecting A added 5 cents reward after a 5-s delay. Selecting B resulted in a variable delay, but added 15 cents. Maximum reward is gained by selecting B, and selecting A is considered the impulsive response. Euthymic BD patients showed significant improvements from their baseline manic scores and no longer differed significantly from the control group in behavioural impulsivity.

In summary, the two stronger studies and one methodologically weaker study found no group differences on impulsivity measures assessing ability to delay gratification. In the papers finding differences, one included patients who were not clearly euthymic, and findings may represent increased impulsivity due to the participants' mood state. The other, Ibanez et al. (2012), had a small sample size and only *post hoc* analyses revealed any group differences when specific blocks of the IGT were combined. However, their finding and the estimated effect sizes provide tentative evidence that euthymic BD patients are less able to delay gratification than controls, although the main differences happened later during the task, potentially indicating a learning deficit or fluctuating response over time.

There is insufficient evidence to draw strong conclusions about the performance of euthymic BD patients on their ability to delay gratification tasks. As well as the limited number of studies, many of the tasks assessing this concept rely on other cognitive processes (such as attention and decision making) that may cloud results.

## Discussion

The current review asked whether there is evidence that BD is associated with increased impulsivity even during euthymia, i.e. a potential vulnerability marker. The studies using self-report measures suggest impulsivity is a trait of those who experience BD rather than only ‘state’ related. Most studies - including the methodologically strong ones - found that euthymic BD patients scored higher than controls on all subscales of the BIS, involving large effect sizes. The mean BIS total scores for over half the studies were also at levels indicating highly impulsive individuals. Additionally, in the longitudinal study by Strakowski et al. (2010), BIS scores did not significantly differ during mania, suggesting stability in the levels of impulsivity.

The performance on behavioural tasks did not indicate the same pattern. Few RI studies detected differences between euthymic BD patients and controls, and there is therefore limited evidence of impulsive behaviour on RI tasks. However, exploration of the effect sizes of group differences suggested that a number of studies with small samples obtained medium effects despite not finding significant group differences, i.e. potentially being a problem of insufficient power. With regards to the ability to delay gratification, the two methodologically stronger studies found little evidence of group differences. However, the small number of studies and paradigms used to assess delay of gratification make it hard to draw firm conclusions.

One question is whether we can conclude that the tentative evidence of increased impulsivity reported above indicates that this is a vulnerability marker for BD. There seems to be an association between course of BD and impulsivity. Kolur et al. (2006) reported that those with a history of two mood episodes made more commission errors than those with only one previous episode. Bora et al. (2007) reported an association between impulsivity and a history of psychotic symptoms in BD. The direction of this relationship is difficult to establish. Rather than impulsivity being a trait marker of those with BD, there may be a detrimental effect on impulsivity from experiencing repeated mood episodes, and this deficit may persist in euthymia. Alternatively, those who are more impulsive may have a more severe course of illness. Research into impulsivity in symptomatic and ‘at risk’ for mania groups also suggests that impulsivity is related to a worsening course of illness and greater chronicity of symptoms. Swann et al. (2009a) found those with more previous mood episodes have higher BIS total scores. Kwapil et al. (2000) reported in their longitudinal study of those at risk for mania that those scoring highly on a self-report impulsivity measure experienced greater rates of BD and poorer overall adjustment to the illness. This gives some support to impulsivity as a trait which may lead to a more severe course of illness.

A related question is the ‘state’ versus ‘trait’ discussion. With respect to behavioural measures, the conceptualisation of state versus trait is not clear cut. Some suggest that behavioural measures are state measures as they assess performance in that moment. However, other researchers (e.g. Najt et al. 2007) regard such measures as trait measures when assessed in a stable mood. Based on the reviewed evidence, we suggest impulsivity, incorporating all recognised facets, has not been identified as a stable trait of euthymic BD patients. Rather, some facets of impulsivity measured via self-report continue to be elevated, whilst RI manifestation of impulsivity may have a more state than trait relationship to BD.

There are a number of possible explanations for the difference in results between self-report and behavioural measures. We already alluded to some, such as potential problem with insufficient power of many studies using behavioural paradigms. Furthermore, self-reports of impulsivity might not be measuring a stable trait in this population. Those with BD may not use the same frame of reference when completing measures such as the BIS as controls. Someone with a history of (hypo)mania may answer the question ‘I do things without thinking’ by considering experiences from their manic episodes. Strakowski et al. (2010) identified this problem and acknowledges that ‘separating affective symptoms from a bipolar individual's “usual self” is not always straight-forward’. It is possible that rather than representing on-going impulse-control difficulties, the BIS reflects the person's view about their general functioning regarding impulsivity, including when in a mood episode. A further explanation for the discrepancy in results could be that most studies in this review used behavioural paradigms that were not primarily designed to measure impulsivity. For example, CPT is primarily an attention paradigm and the *Iowa Gambling Task* is commonly referred to as a decision-making task. Any potential deficits in these processes may influence the outcomes linked to impulsivity. The lack of support for impulsivity as a trait of individuals with BD from these paradigms may therefore represent questionable construct validity rather than a true lack in group differences in relation to the facet of impulsivity assessed. Another consideration is that self-report and behavioural measures of impulsivity may be measuring different constructs, which are often not correlated with each other (e.g. Reynolds et al. 2006; Stanford et al. 2009). It seems essential in future research to refer more specifically to the processes and functions in question instead of impulsivity.

Lastly and related to the previous issue about looking more in depth into the processes and functions of impulsivity, impulsive behaviours could be activated by specific emotional states in euthymic BD patients. For example, Johnson et al. (2013) found that an increased risk for mania was associated with a tendency to behave impulsively when in elevated mood. Mansell and Lam (2006) also found that euthymic BD patients were less likely to follow advice following positive mood induction. These results suggest that mood elevations do not need to reach the level of mania for impulsive behaviour to be activated. Impulsivity therefore may be a state-related characteristic of those who experience BD.

Before drawing the final conclusions, some limitations of this review should be noted. Although we systematically searched for data, we cannot rule out that studies have been missed or non-significant results not been published. The application of the grid was done by one reviewer, and no reliability assessment was conducted. However, given the clear categories used, it seems unlikely that this would have changed the results. Furthermore, the boundaries of the concept of impulsivity are not clear so that every selection of tools and measures could be judged as arbitrarily. We tried to be as explicit as possible to allow an evaluation of the measures we included. We see it as strength that contrary to any previous reviews on impulsivity and BD, we have considered relevant studies that did not explicitly investigate impulsivity but presented the indices which have been interpreted as indicating impulsivity. Lastly, a small number of the papers had samples that were likely to include subsyndromal patients. If impulsivity is activated in such subsyndromal states, this could have influenced the results. However, it is unlikely that this explains the difference between euthymic patients and controls on self-report impulsivity measures, as the studies with the most stringent definitions of euthymia did find significant group differences (e.g. Ekinci et al. 2011; Strakowski et al. 2010).

## Conclusions

Despite the limitations, we believe there is significant evidence from the studies using self-report impulsivity measures that scores remain elevated during the euthymic stage of BD. There is therefore sufficient evidence of some facets of impulsivity persisting in the self-perception of patients during euthymia. It is less clear what the direction of this relationship might be. However, it is still not clear if elevated impulsivity is a vulnerability factor for developing BD existing before the onset of the disorder or whether elevated self-reported impulsivity is a consequence of the disorder, a residual symptom or a reference to their previous mood-related behaviour.

Euthymic BD patients have not been clearly identified as more impulsive on behavioural measures assessing RI. However, this might rather reflect methodological issues of the studies. There is tentative evidence that those with more severe symptoms may continue to perform more impulsively on RI measures even when euthymic. Looking at the ability to delay gratification, much more research is needed before drawing any conclusions. Future research would also benefit from including those at risk of developing BD. Some evidence exists and provides tentative evidence of impulsivity as a vulnerability marker for BD (e.g. Kwapil et al. 2000; Fulford et al. 2008; Giovanelli et al. 2013). However, there is insufficient literature to draw conclusions about which specific facets of impulsivity are the traits of individuals who go on to develop BD, rather than a consequence of the illness.
